# Mitochondrial DNA Corroborates the Genetic Variability of *Clarias* Catfishes (Siluriformes, Clariidae) from Cameroon

**DOI:** 10.3390/life13051068

**Published:** 2023-04-22

**Authors:** Shantanu Kundu, Piyumi S. De Alwis, Jerome D. Binarao, Soo-Rin Lee, Ah Ran Kim, Fantong Zealous Gietbong, Myunggi Yi, Hyun-Woo Kim

**Affiliations:** 1 Department of Marine Biology, Pukyong National University, Busan 48513, Republic of Korea; 2 Research Center for Marine Integrated Bionics Technology, Pukyong National University, Busan 48513, Republic of Korea; 3 The Ministry of Livestock, Fisheries and Animal Industries (MINEPIA), Yaoundé 00237, Cameroon; 4 Department of Biomedical Engineering, Pukyong National University, Busan 48513, Republic of Korea

**Keywords:** walking catfish, DNA barcoding, haplotypes, phylogeny, genetic diversity

## Abstract

The airbreathing walking catfish (Clariidae: *Clarias*) comprises 32 species that are endemic to African freshwater systems. The species-level identification of this group is challenging due to their complex taxonomy and polymorphism. Prior to this study, the biological and ecological studies were restricted to a single species, *Clarias gariepinus*, resulting in a biased view of their genetic diversity in African waters. Here, we generated the 63-mitochondrial Cytochrome c oxidase subunit 1 (COI) gene sequences of *Clarias camerunensis* and *Clarias gariepinus* from the Nyong River in Cameroon. Both *C. camerunensis* and *C. gariepinus* species maintained adequate intra-species (2.7% and 2.31%) and inter-species (6.9% to 16.8% and 11.4% to 15.1%) genetic distances with other *Clarias* congeners distributed in African and Asian/Southeast Asian drainages. The mtCOI sequences revealed 13 and 20 unique haplotypes of *C. camerunensis* and *C. gariepinus,* respectively. The TCS networks revealed distinct haplotypes of *C. camerunensis* and shared haplotypes of *C. gariepinus* in African waters. The multiple species delimitation approaches (ABGD and PTP) revealed a total of 20 and 22 molecular operational taxonomic units (MOTUs), respectively. Among the two *Clarias* species examined, we found more than one MOTU in *C. camerunensis*, which is consistent with population structure and tree topology results. The phylogeny generated through Bayesian Inference analysis clearly separated *C. camerunensis* and *C. gariepinus* from other *Clarias* species with high posterior probability supports. The present study elucidates the occurrence of possible cryptic diversity and allopatric speciation of *C. camerunensis* in African drainages. Further, the present study confirms the reduced genetic diversity of *C. gariepinus* across its native and introduced range, which might have been induced by unscientific aquaculture practices. The study recommends a similar approach to the same and related species from different river basins to illuminate the true diversity of *Clarias* species in Africa and other countries.

## 1. Introduction

The airbreathing catfish family, Clariidae (order Siluriformes), comprises 117 species under 16 genera [1]. They are primarily freshwater species and distributed in Africa, Syria, and Southern and Western Asia (Philippines to Java). Owing to their ability to walk on land, these catfishes are also known as walking catfish. Among all extant Clariidae catfishes, the genus *Clarias* Scopoli, 1777, is the most species-rich group, with 63 valid species described so far [1,2]. With the increasing rate of *Clarias* species descriptions from Asian and Southeast Asian countries [3], a new species, *Clarias monsembulai* Bernt and Stiassny, 2022, was recently described from the African continent after 42 years of the latest species (*Clarias agboyiensis* Sydenham 1980) discovery [4]. The species-level identification of this group is challenging due to complex taxonomy and sexual polymorphism [5,6,7,8]. In addition to commercial value as a nutritious food source and aquarium decoration, this species group maintains a predator–prey role in food webs and ecosystems. However, this group of species confronts several threats, such as habitat shifting and alteration, massive over-exploitation, wastewater effect, and invasion of alien species [9,10,11,12,13,14].

It is imperative to know the genetic diversity of any biodiversity elements to monitor and conserve in their ecosystems beyond any political boundary to promote the Convention on Biological Diversity—Nagoya Protocol, on access to genetic resources and equitable sharing of benefits arising from their use (https://www.cbd.int/abs/, accessed on 4 March 2023). The genetic data have become an increasingly valuable tool in informing conservation and management strategies for freshwater fishes and other animals [15,16]. By providing information on population structure, genetic diversity, connectivity, and hybridization, genetic data can help inform management decisions and ensure the long-term sustainability of many important fishes including *Clarias* [17,18,19,20]. In addition to the high species diversity of *Clarias*, only three species (*Clarias batrachus* Linnaeus, 1758, *Clarias gariepinus* Burchell, 1822, and *Clarias macrocephalus* Günther, 1864) have been repeatedly studied on different aspects, resulting in a limited view of their biological and ecological perspectives [21,22,23,24,25]. Microsatellite markers and nuclear and mitochondrial gene sequences were utilized to reveal the phylogenetic relationship, population structure, and diversification of *Clarias* species [26,27,28,29,30]. In order to improve the in-depth phylogenetic relationship, the genome sequences of *C. batrachus* were also generated and analyzed [31,32,33]. Further, with the advancement of molecular tools, environmental DNA was also examined to track the diversity of *Clarias* species [34]. In addition, several small- to large-scale DNA barcoding attempts have been made to illuminate the genetic diversity of catfishes around the world, including in Africa [35,36,37,38]. A recent study generated the first mitochondrial genome of *C. camerunensis* and elaborated the diversification of *Clarias* species in Africa and the Asian continent [39].

However, the genetic diversity of *C. camerunensis* Lönnberg, 1895, and *C. gariepinus* is poorly known from Cameroon waters. *Clarias camerunensis* was described from the Sanaga River, Cameroon, and distributed in Western and Western-Central Africa (Togo south to the Democratic Republic of Congo (DRC), including the Congo River basin). Although few gene sequences were generated from the different drainage systems of the DRC, the Republic of Congo (RC), and Nigeria, the genetic diversity is unknown from its type locality in Cameroon. *Clarias gariepinus* has a pan-African distribution and is well studied throughout African drainages, except for Cameroon. In the recent past, the Republic of Korea agreed to a cooperation framework for members of the Congo Basin Forest Partnership to promote the sustainable management of forest ecosystems in Central Africa (https://pfbc-cbfp.org/news-partner/Welcome-Korea-CBFP.html (accessed on 7 April 2023). To promote molecular techniques in systematics and biodiversity research in African countries, the present study aimed to generate DNA barcode data for two catfishes, *C. camerunensis* and *C. gariepinus,* from Cameroon waters and compared their genetic diversity with other known distant populations. This genetic information will aid in the speedy and accurate identification of *Clarias* species, as well as the conservation and sustainable utilization of *Clarias* species in aquaculture by determining their genetic diversity in African drainage systems.

## 2. Material and Methods

### 2.1. Sampling and Identification

**Figure 1:**
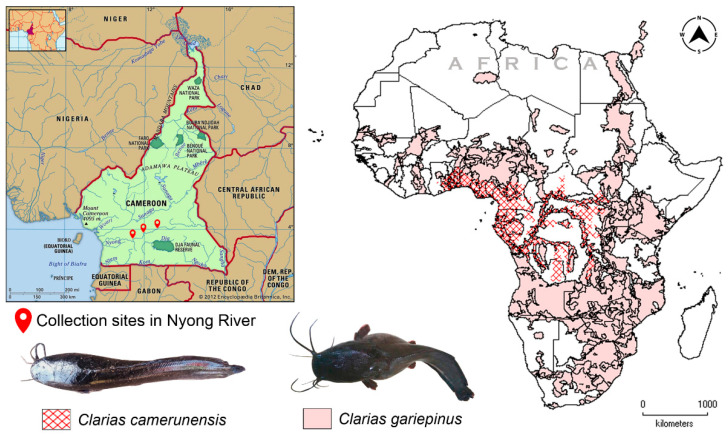
Map showing the collection localities of *C. camerunensis* and *C. gariepinus* from Nyong River in Cameroon (marked by the red pins). The Physical features of Cameroon map were acquired from Encyclopedia Britannica (https://www.britannica.com/place/Cameroon#/media/1/90925/61979, accessed on 4 March 2023). The IUCN range distribution of *C. camerunensis* and *C. gariepinus* in African continent. Map was prepared using .shp files and acquired from IUCN and DIVA-GIS platform. Species photographs were captured by Fantong Zealous Gietbong.

A total of 63 *Clarias* specimens were collected from three different localities (3.760820° N 12.173112° E, 3.764950° N 12.246810° E, 3.815711° N 12.346524° E) in the Nyong River from Cameroon during July 2019 to November 2020 (Figure 1). The specimens were captured by using a standard cast net and euthanized with MS-222 (200 mg/L). The specimens were identified as *C. camerunensis* (n = 50) and *C. gariepinus* (n = 13) based on the major taxonomic keys [7,8]. The muscle tissue was aseptically excised from the ventral thoracic region of each specimen and preserved in 70% ethanol for molecular experiments. The voucher specimens were fixed in 10% formaldehyde for long-term preservation at Fisheries and Animal Industries (MINEPIA), Cameroon. The tissue samples and genomic DNA were stored in the Department of Marine Biology at Pukyong National University, Busan, South Korea. No prior permission was required for sampling, and the host institutions approved the molecular data generation and analyses.

### 2.2. DNA Extraction, PCR Amplification, and Sequencing

Approximately 100 mg of muscle tissue was taken aseptically and added to 700 μL lysis buffer, 60 µL SDS, and 40 µL of Proteinase K in a 1.5 mL centrifuge tube. The genomic DNA was extracted using the AccuPrep^®^ Genomic DNA Extraction Kit (Bioneer, Daejeon, Republic of Korea) with standard protocol. The mtCOI partial gene fragment (~650 bp) was amplified using the published primer pairs (BCL and BCH) [40]. The amplification reactions were performed with a total volume of 30 µL (1× PCR buffer, 10 pmol of each primer, 2.5 mM of dNTPs, 1 U of Taq polymerase, and 1 µL of template DNA) using a TaKaRa PCR Thermal Cycler Dice^®^ Gradient (Takara Korea Biomedical Inc., Seoul, Republic of Korea) with the standard thermal profile. The amplified PCR products were visualized in 1.5% agarose gel and ethidium bromide stain (10 mg/mL) compared with 100 bp DNA ladder. The targeted bands were cut out from the gel and purified using AccuPrep^®^ PCR/Gel Purification Kit (Bioneer, Daejeon, Republic of Korea) following the manufacturer’s protocols. The amplicons were further amplified with the BigDye(R) Terminator v3.1 Cycle Sequencing Kits (Applied Biosystems) in DNA Engine Tetrad 2 Peltier Thermal Cycler (BIO-RAD) and sequenced bi-directionally in an automated sanger sequencer (96 capillaries ABI PRISM 3730XL Analyzer) at Macrogen (https://dna.macrogen.com/ (accessed on 7 April 2023) (Daejeon, Republic of Korea).

### 2.3. Sequence Quality Check

The low-quality regions were trimmed from both 3’ and 5’ ends of forward and reverse sequences using the software SeqScanner version 1.0 (Applied Biosystems Inc., CA, USA). Both forward and reverse complements of reverse sequences were aligned through ClustalX software to make a consensus sequence [41]. The consensus sequences were further reviewed through nucleotide BLAST search (https://blast.ncbi.nlm.nih.gov (accessed on 7 April 2023) and ORF finder (https://www.ncbi.nlm.nih.gov/orffinder/ (accessed on 7 April 2023) to avoid the insertion/deletions and confirm the appropriate amino acid array. The final barcode sequences were submitted to GenBank via the Bankit submission tool (https://www.ncbi.nlm.nih.gov/WebSub/ (accessed on 7 April 2023).

### 2.4. Dataset Construction and Analyses

To examine the genetic diversity of *C. camerunensis* and *C. gariepinus*, three different datasets were constructed for phylogenetic and haplotypic analyses. In the first dataset, a total of 63 sequences (50 generated in this study and 13 acquired from GenBank) of *C. camerunensis* were analyzed to examine the haplotype diversity and network construction. To estimate the haplotypic diversity of *C. gariepinus*, the second dataset was constructed with 13 generated and 174 database sequences. The number of haplotypes and haplotype diversity (Hd) were estimated by using DnaSP v4.10.9 [42]. The TCS networks of all haplotypes were constructed in Popart [43,44]. In the third dataset, 287 sequences (63 generated and 224 databases) of 18 *Clarias* species were acquired from GenBank. The DNA sequences (MG824585, JF510512, and HM882796) of *Clarotes laticeps* (Siluriformes: Claroteidae) were used in the dataset as an out-group. The first dataset was aligned using the ClustalX program, and the best-fit model was estimated through PartitionFinder version 1.1.1 and Mr. MODELTEST version 2, with the lowest BIC score [45,46]. Because the PartitionFinder found the same model (GTR + I + G) for all three codon positions, we did not partition the data further. The Bayesian Inference was built in Mr. Bayes v3.1.2 by choosing nst = 6 for GTR + G + I model and four (one cold and three hot) MCMC, and it was run for 1,000,000 generations with 25% burn-in with tree saving every 100 generations [47]. The MCMC analysis was applied to generate the convergence metrics until the standard deviation of split frequencies arrived at 0.01 and the PSRF for all parameters neared 1.0. The BA phylogeny was further illustrated using the web-based iTOL tool (https://itol.embl.de/ (accessed on 7 April 2023) [48]. The genetic distances were calculated using Kimura 2 parameter (K2P) by MEGA11 [49]. To check the molecular operational taxonomic units (MOTUs), two species delimitation methods, Automatic Barcode Gap Discovery (ABGD) and Poisson Tree Process (PTP) analyses, were further applied for the third dataset [50,51]. Both ABGD and bPTP analyses were performed by using iTaxoTools 0.1 tool with default parameters [52]. Both Kimura (K80) and Jukes–Cantor (JC69) models were tested for ABGD analysis to estimate the MOTUs. The maximum-likelihood tree was constructed in MEGA11, and the unrooted Newick format was utilized for the PTP analysis.

## 3. Results

### 3.1. Genetic Divergence and Haplotype Distribution

The generated mtCOI sequences (633 bp) of *C. camerunensis* and *C. gariepinus* were contributed to the global GenBank database and acquired the accession numbers (OP420808 to OP420857) and (OP555273 to OP555285), respectively. The generated sequences revealed 99–100% similarity with the GenBank sequences of *C. camerunensis* and *C. gariepinus* from African waters. The overall mean genetic divergence was 7.6% in the third dataset of the *Clarias* species. Both *C. camerunensis* and *C. gariepinus* showed 2.7% and 2.31% intra-species genetic distances, respectively. An unexpectedly high intra-species genetic distance (6.6%) was depicted in the GenBank sequences of *Clarias angolensis*, which needs further verification by generating more data from its range. The third dataset also depicts high inter-species genetic distance (17.3%) between all 18 *Clarias* species. Both *C. camerunensis* and *C. gariepinus* maintained 6.9% to 16.8% and 11.4% to 15.1% genetic distances from other *Clarias* species.

**Figure 2:**
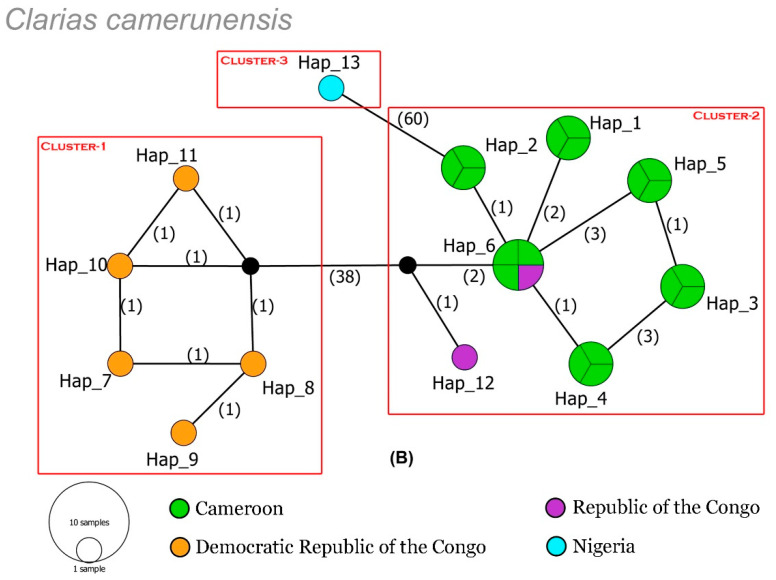
The TCS haplotypic network shows the relationship among all the haplotypes of *C. camerunensis* from different drainage systems in Africa. Circle sizes are proportional to the haplotype frequencies. The number of mutations is represented by the number in parentheses. The median vectors (hypothetical haplotypes) are denoted by black circles.

**Figure 3:**
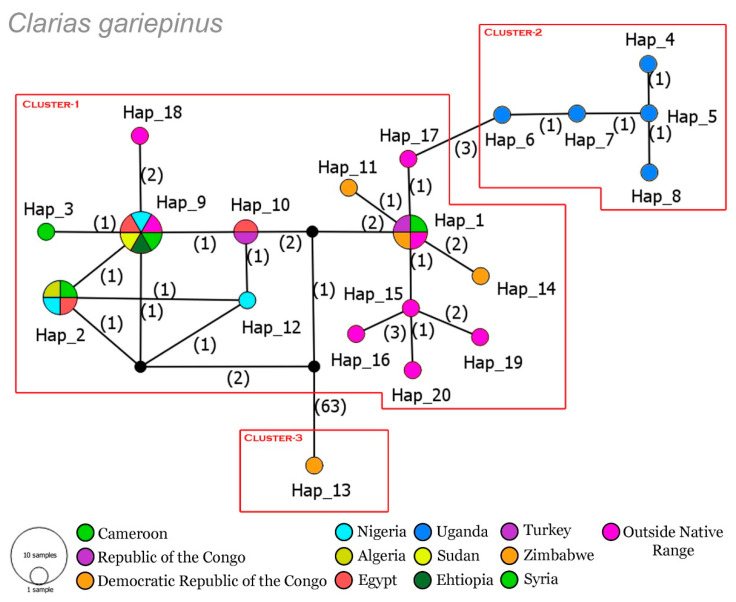
The TCS haplotypic network demonstrates the relationship among all the haplotypes of *C. gariepinus* from different drainage systems in Africa and Asia. Circle sizes are proportional to the haplotype frequencies. The number of mutations is represented by the number in parentheses. The black circles denote the median vectors (hypothetical haplotypes).

The dataset of *C. camerunensis* revealed 13 haplotypes with 86 segregating sites, 48 parsimony informative sites, haplotype diversity (Hd) = 0.8920, and nucleotide diversity (π) = 0.2830. The TCS network depicted three distinct clusters of *C. camerunensis* collected from different drainage systems of four African countries viz., Nigeria, Cameroon, DRC, and RC (Figure 2). Cluster 1 and Cluster 3 were restricted by the specimens collected from DRC and Nigerian drainage systems, respectively. However, Cluster 2 was represented by the specimens from Cameroon and RC drainages with shared haplotypes. The *C. gariepinus* dataset represents 20 haplotypes with 81 segregating sites, 13 parsimony informative sites, haplotype diversity (Hd) = 0.8539, and nucleotide diversity (π) = 0.2578. Most of the sequences of *C. gariepinus* revealed shared haplotypes throughout different drainage systems in Africa. The TCS network also represents three clusters in *C. gariepinus*. Cluster 1 represents the specimens collected from different drainage systems in native and outside ranges. However, Cluster 2 represents the specimens restricted to Uganda drainages, and Cluster 3 represents a single sequence generated from Zimbabwe rivers, which needs further investigation (Figure 3).

### 3.2. MOTU Estimation and Phylogenetic Relationship

The multiple species delimitation methods (ABGD and PTP) showed 22 and 20 MOTUs, respectively, in the studied dataset. More than one MOTU was detected in *C. camerunensis,* corresponding to different drainage systems in DRC, Cameroon+ RC, and Nigeria. Most of the sequences of *C. gariepinus* generated from native and distant localities in non-native regions revealed a single MOTU. However, a single database sequence (OL311814) generated from Zimbabwe showed a distinct MOTU, which needs further verification. Among other African *Clarias*, most of the species revealed single MOTU, except *C. macrocephalus* and *C. gabonensis* with multiple MOTUs, which requires further investigation. Further, the other two species (*C. jaensis* and *C. buthupogon*) showed separate MOTUs in ABGD and single MOTU in PTP analysis, encouraging further systematics reevaluation from African waters.

**Figure 4:**
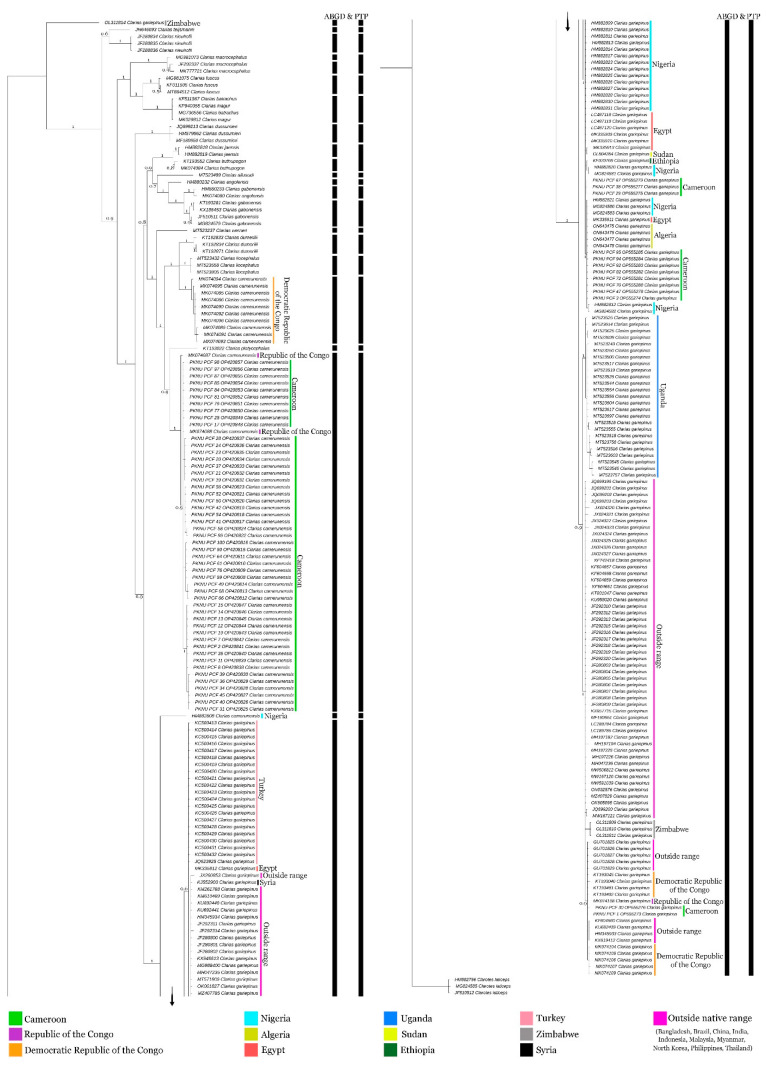
The Bayesian phylogeny based on mtCOI sequences showed distinct clustering of *Clarias camerunensis* and *Clarias gariepinus* from other congeners. The generated sequences in this study are remarked with their DNA banking numbers ‘PKNU PCF’. The MOTUs estimation by multiple species delimitation methods (ABGD and PTP) are presented by black side bars.

The Bayesian (BA) phylogeny showed distinct clades of all *Clarias* species with high posterior probability supports. Both the African and Asian clades cohesively clustered in the present topology (Figure 4). The specimens of *C. gariepinus* collected from diverse drainage systems in Africa and outside revealed monophyletic clustering in the present topology. However, the specimens of *C. camerunensis* were revealed to be paraphyletic, with three separate clades being recovered, one with specimens from Cameroon and RC drainages, the other with specimens from DRC drainage, and the third one with specimens from Nigerian drainage. Considering the limited sample size of *C. camerunensis* covering a smaller number of riverine systems in the known range, this preliminary genetic information indicates high genetic variability, the formation of distinct clades in BA phylogeny, and strong haplotype structuring, evidenced by the presence of a distinct population of *C. camerunensis* in African waters. Further, the reduced genetic variation, monophyletic clade, and shared haplotyping network depicted the coalesced population of *C. gariepinus* in different freshwater systems in Africa. The sequences generated from outside of the native range (Bangladesh, Brazil, China, India, Indonesia, Malaysia, Myanmar, North Korea, Philippines, and Thailand) also depicted close clustering with the sequences generated from African waters. However, the single sequence of *C. gariepinus* (Accession No. OL311814) generated from Zimbabwe water, distantly cladded which needs further re-examination from its voucher.

## 4. Discussion

Biodiversity is an asset of any nation that should be conserved with high priority. Thus, recognizing the genetic diversity of native species is crucial for various management action plans for the conservation of the ecosystem. The biogeography of Cameroon is unique, with 13 terrestrial ecoregions, 1 marine ecoregion, and 1 pelagic province [53,54]. The native biodiversity of Cameroon is safeguarded by 39 terrestrial (51,538.0 km^2^) and 2 marine (1601.6 km^2^) protected areas. This country is often regarded as a miniature of Africa due to its diverse landscape from coastal areas to mountains with savanna and rainforest [55,56]. Cameroon ranks fifth for faunal diversity in Africa and accommodates about 564 freshwater fish species in different river basins [1,2]. Most interestingly, the biogeography and structure of these river basins play an important role in predicting the diversification of freshwater fishes [57]. Globally, fish diversity is intensely threatened due to the overexploitation, habitat destruction, and effects of climate change in the world, particularly in Cameroon [58,59]. Considering the high ichthyofaunal diversity in Cameroon, the assessment of genetic diversity is important apart from the taxonomic assessment. A high genetic variability/cryptic diversity was observed in African fishes earlier and correlated to their evolution linked with geotectonic events and climate changes [23,60].

The present results also indicate that *C. camerunensis* populations are geographically isolated with restricted gene flow in African waters. This restricted haplotypic distribution in different riverine systems may enlighten their independent evolution and local adaptations. It is evidenced that biogeographic variation directly affects the population structure of any species by accumulating genetic mutation through their mobility, colonization, and isolation [61]. The continental diversification and geographic barriers have triggered the radiation and independent colonization of fish species in different biodiversity hotspots and river basins [62,63,64]. The surveyed Nyong River is flowing west and south from its source to the east of the Abang–Mbang tropical rainforest of east-central Cameroon and joins the Gulf of Guinea in the Atlantic Ocean. Although the headwaters of the Nyong River lie close to the Congo River basin, a significant genetic divergence was observed in *C. camerunensis* collected from this riverine system and compared with the specimens from the Inkisi and Luki River flows in the DRC. Both the Inkisi and Luki Rivers in DRC are the south bank tributaries of the Congo River, which may constitute different genetic makeup for their biotic elements. Further, the Kouilou–Niari River flows from the Sounda gorges to the coastal region of Kouilou through Niari Valley and harbors unparallel freshwater biodiversity in RC. The high genetic variability in *C. camerunensis* probably reveals past or ongoing allopatric speciation or the presence of cryptic diversity in African waters. Further, *C. gariepinus* is well established in aquaculture and introduced in several riverine systems in Africa and other countries [9,10,12]. In the recent past, the diversification of *C. gariepinus* has been illustrated, linked with the evolution of drainage basins in Africa [23]. The earlier genetic investigation with mitochondrial Cytb gene (approximately 1140 bp) and microsatellite genotyping clearly segregated different populations of *Clarias* species with high genetic diversity in different provinces on the African continent [23]. The present genetic assessment is congruent with the earlier hypothesis and supported the unparallel distribution of this *Clarias* species [23]. The present analyses support that the human-induced translocations and aquaculture practices of *C. gariepinus* have left genetic diversity and sharing haplotypes between geographically distant areas.

Sustainable aquaculture practices always require the assessment of genetic diversity of both wild and farmed populations [65,66]. This genetic information is essential for selective breeding, ensuring gene flow, and diminishing inbreeding depression for long-term farming [67,68,69]. It also helps to eliminate the outbreeding, genetic homogenization, and competition issues; therefore, the genetic specificity of domesticated stocks can be advantageous for developing aquaculture management strategies [70,71]. *C. camerunensis* is not yet significant in aquaculture; however, if this species is targeted for aquaculture, a lack of understanding of its genetic variability, unscientific breeding, and transmigration may naturally lead to genetic mixing and depletion of separate lineages [72,73]. To achieve selective breeding, the genome-wide genetic diversity estimation has been attempted for many commercially important fishes globally [74,75]. Therefore, the present knowledge on the genetic diversity of *C. camerunensis* discovered in the current research from different African river systems is critical before this species is welcomed into aquaculture practices.

## 5. Conclusions

The mtCOI gene successfully delineated both African and Asian *Clarias* species in this study. Our results highlight the high genetic variability and unique haplotypes and MOTUs of *C. camerunensis* and reveal the allopatric speciation or presence of cryptic diversity in African waters. The present analyses also support the coalesced distribution of *C. gariepinus* in African and Asian waters. We propose a large-scale integrated assessment of morphology, genetics, and spatial ecology to monitor and protect *Clarias* diversity. To minimize inbreeding and the genetic erosion of several indigenous fish species for profitable aquaculture in varied habitats in Africa, we advocate developing genome-wide population genetic structure and subsequent planning.

## Data Availability

The specimens were vouchered at Fisheries and Animal Industries (MINEPIA), Cameroon. The tissue samples and genomic DNA were stored in the Department of Marine Biology, at Pukyong National University, Busan, South Korea. The nucleotide sequence data that support the findings of this study are available in GenBank of NCBI at [https://www.ncbi.nlm.nih.gov] under the accession no. OP420808 to OP420857 and OP555273 to OP555285.
